# Co-Clinical Trials: An Innovative Drug Development Platform for Cholangiocarcinoma

**DOI:** 10.3390/ph14010051

**Published:** 2021-01-11

**Authors:** Brinda Balasubramanian, Simran Venkatraman, Kyaw Zwar Myint, Tavan Janvilisri, Kanokpan Wongprasert, Supeecha Kumkate, David O. Bates, Rutaiwan Tohtong

**Affiliations:** 1 Graduate Program in Molecular Medicine, Faculty of Science, Mahidol University, Bangkok 10400, Thailand; balasubramanian.bri@student.mahidol.edu (B.B.); simran.ven@student.mahidol.ac.th (S.V.); kyawzwar.myn@student.mahidol.edu (K.Z.M.); 2 Department of Biochemistry, Faculty of Science, Mahidol University, Bangkok 10400, Thailand; tavan.jan@mahidol.ac.th; 3 Department of Anatomy, Faculty of Science, Mahidol University, Bangkok 10400, Thailand; kanokpan.won@mahidol.ac.th; 4 Department of Biology, Faculty of Science, Mahidol University, Bangkok 10400, Thailand; supeecha.kum@mahidol.ac.th; 5 Division of Cancer and Stem Cells, School of Medicine, Biodiscovery Institute, University of Nottingham, Nottingham NG7 2RD, UK; David.Bates@nottingham.ac.uk

**Keywords:** cholangiocarcinoma, co-clinical trials, targeted therapy, clinical trials, precision medicine

## Abstract

Cholangiocarcinoma (CCA), a group of malignancies that originate from the biliary tract, is associated with a high mortality rate and a concerning increase in worldwide incidence. In Thailand, where the incidence of CCA is the highest, the socioeconomic burden is severe. Yet, treatment options are limited, with surgical resection being the only form of treatment with curative intent. The current standard-of-care remains adjuvant and palliative chemotherapy which is ineffective in most patients. The overall survival rate is dismal, even after surgical resection and the tumor heterogeneity further complicates treatment. Together, this makes CCA a significant burden in Southeast Asia. For effective management of CCA, treatment must be tailored to each patient, individually, for which an assortment of targeted therapies must be available. Despite the increasing numbers of clinical studies in CCA, targeted therapy drugs rarely get approved for clinical use. In this review, we discuss the shortcomings of the conventional clinical trial process and propose the implementation of a novel concept, co-clinical trials to expedite drug development for CCA patients. In co-clinical trials, the preclinical studies and clinical trials are conducted simultaneously, thus enabling real-time data integration to accurately stratify and customize treatment for patients, individually. Hence, co-clinical trials are expected to improve the outcomes of clinical trials and consequently, encourage the approval of targeted therapy drugs. The increased availability of targeted therapy drugs for treatment is expected to facilitate the application of precision medicine in CCA.

## 1. Introduction

Cholangiocarcinoma (CCA) is a group of malignancies that originate from the biliary tract with increasing worldwide incidence in the last decade [1]. The socioeconomic burden of CCA is severe, particularly in the Southeast Asia. Thailand has the highest CCA incidence, where it is almost 100 times more prevalent (85 per 100,000) than Western countries (0.8–2 in 100,000) [2]. The alarming mortality rate of 14%, which roughly translates to 20,000 deaths every year, makes CCA a cause for concern [3]. Treatment is challenging as it is usually asymptomatic in the early stages and diagnosed in the advanced stages, with dismal prognosis and a discouraging 7–20% 5-year survival rate [1,4,5]. Surgical resection and liver transplant, the only form of treatment with curative intent, are technically challenging and require specially trained personnel [6]. The median survival time following surgical resection is 15 months; the 3-year survival rate of 35 to 50% is achieved almost exclusively in patients with a negative histological margin at the time of surgery [7,8,9]. Nevertheless, even after surgical resection, the cancer recurrence rate is high [10]. Adjuvant chemotherapy after surgical resection has been expected to overcome recurrence, yet they do not lengthen the overall survival [11]. Moreover, surgical resection is limited to patients diagnosed in the early stages. CCA patients in advanced stages, that present with local invasion or distant metastasis, are generally inoperable, restricting their treatment options to solely palliative chemotherapy [4,12]. The overall survival for patients with unresectable tumor is just under 12 months from diagnosis [3].

CCA is highly heterogenous in terms of tumor pathology, genetics, primary origin, risk factors, epidemiology, and clinical features [2,13,14], all of which further complicate treatment. Generally, CCA is classified into intrahepatic (iCCA) and extrahepatic (perihilar or distal) subtypes based on the anatomical location of primary [2,15,16]. In clinical trials, the different CCA subtypes are generally pooled together, because, the heterogeneity within the subtypes is unclear until recent advances in molecular characterization techniques such as next-generation sequencing (NGS) [17]. Genetic profiling studies using NGS have elucidated the distinct molecular profiles present in intrahepatic and extrahepatic CCA [18]. Altogether, the inadequate patient stratification in clinical trials often results in poor outcomes and trial failure. Currently, physicians are compelled to treat CCA patients with the premise “one size fits all” due to the lack of available therapeutic alternatives. Precision medicine in cancer, also known as “precision oncology”, considers the heterogeneity of cancer and thus abandoning the “one size fits all” premise. It combines different aspects of molecular profiling to appropriately inform diagnosis, prognosis, and thereby customizing treatment for patients, individually [19]. This is practiced in high-income countries (HICs) for prevalent cancers such those of the lung, breast, etc., to predict treatment outcomes in patients [20]. However, this is yet to be practiced in CCA patients, particularly, in low and middle-income countries (LMICs) in Southeast Asia. Increasing evidence supports the use of guided targeted therapies to improve overall survival for CCA patients [21,22,23,24,25]. Therefore, considering the heterogeneity of CCA, treatment should be tailored to individual patients with targeted therapy.

Molecular and mutational profiling studies using NGS have elucidated a number of biomarkers exclusive for CCA [18,26] and since then, several targeted therapy drugs have emerged in CCA in recent years to meet the urgent demand for novel therapeutic options. Despite the increasing number of clinical trials in CCA, there is still a lack of targeted therapy drugs available for treatment. A majority of clinical trials with targeted therapy drugs fail to meet their endpoint objectives, mainly because a mixed cohort of patients are recruited to the study, as a consequence, the test drugs result in poor outcomes and fail to get regulatory approval [27]. This highlights the urgency of developing targeted therapy drugs for CCA and the need for an effective clinical trial platform to expedite the process. 

In this review, we propose that the implementation of co-clinical trials will expedite the approval of targeted therapy drugs in CCA by improving trial outcomes. The primary objective of co-clinical trials is to conduct pre-clinical and clinical studies in parallel to allow for real-time integration of data for an effective study design in clinical trial, consequently improving the outcomes for clinical trials. Improved outcomes from clinical trials will encourage the approval of targeted therapy drugs for the selected cohort of CCA patients.

## 2. Current Standard-of-Care in CCA Management

Treatment options for CCA patients are stratified based on disease progression. For patients in early stages, surgery with curative intent followed by adjuvant chemotherapy with capecitabine is the current standard-of-care [28]. Whereas, patients in advanced stages are limited to a combination of gemcitabine and cisplatin, as first-line standard-of-care [29,30]. However, many patients are not well enough to receive aggressive systemic therapy. While some patients may benefit from the current standard-of-care, others fail to respond to first-line chemotherapy, possibly, due to the aggressive and heterogeneous nature of CCA [31]. For such patients, there has been no second-line standard-of-care until the ABC-06 phase III clinical trial in patients with locally advanced and metastatic biliary tract cancers [NCT01926236]. In this randomized clinical trial, the patients were given FOLFOX (a combination of folinic acid, 5-FU, and oxaliplatin) or ASC (i.e., proactive management of biliary obstruction/sepsis, etc.). The median overall survival of the study arm that was treated with FOLFOX was 6.2 months as opposed to the 5.3 months of the standard arm. As this outcome was clinically significant, FOLFOX is now considered as the second-line chemotherapy for patients that were previously treated with gemcitabine and cisplatin. Nevertheless, the difference in the median overall survival between the two study groups is still modest. In addition, the long-term effects in a larger patient sample group are yet to be evaluated. There is increasing evidence that supports the use of guided targeted therapy drugs to overcome resistance to chemotherapy, by accurately treating patients according to their distinct molecular profiles [31]. That said, there is still a paucity of approved targeted therapies available for CCA treatment. Therefore, effective treatment will only be possible once there is an abundance of targeted therapy drugs available to practice precision medicine in CCA. 

## 3. The Current Landscape of Targeted Therapies in CCA

The onset of molecular profiling of tumors using NGS technology has contributed to a better understanding of the distinct genetic profiles in CCA. Several studies have identified potential targetable mutations and pathways for treatment. Moreover, these studies have elucidated the molecular discrepancies between the subtypes of CCA. Mutations in the genes *isocitrate dehydrogenases* (*IDH1* and *2*) and fusions of the *fibroblast growth factor receptor* 2 (*FGFR2*) were found exclusively in iCCA, whereas *Kirsten rat sarcoma viral oncogene homolog* (*KRAS*) were more common in eCCA [32]. Acknowledging this, and combined with the increasing evidence that advocates the use of targeted therapies, the research attention in CCA has driven towards the development of targeted therapy. However, despite the increase in clinical trials investigating targeted therapies, there is still a lack of it to practice precision medicine in CCA. We posit that this is due to the failure of clinical trials in yielding substantial outcomes. The prime reasons for clinical trial failure in CCA are due to inadequate stratifications of patients and a lack of understanding of the underlying mechanisms of drug response and acquired resistance elicited by certain compounds.

Targeted therapy drugs are anticipated to be incorporated into the treatment regimen based on clinical trial outcomes. Currently, inhibitors of *IDH* and *FGFR* are being investigated in clinical trials following encouraging preliminary results for specific cohorts of patients containing *IDH* mutations [NCT02989857] and *FGFR2* fusions [NCT03656536, NCT03773302]. Hence, biomarker driven clinical trials are expected to facilitate drug development, because, in such trials, the patients are stratified according to the oncogenic driver genes expressed and are more likely to respond to targeted therapy [33]. Yet, many other clinical trials involving potential targeted therapy drugs have failed in CCA. Several such clinical trials fail to recruit the patients conforming to the study designs, for example, studies investigating the effect of ceritinib in *ROS, ALK* mutations positive CCA patients were prematurely terminated due to insufficient recruitment of patients [NCT02374489, NCT02638909]. Several other targeted therapy clinical trials have failed to achieve their endpoint objectives due to study design constraints. Vandetanib, a multiple kinases inhibitor, was tried in patients with advanced metastatic CCA and did not improve progression free survival [NCT00753675] [34]. As the trial was randomized and patients were not stratified based on molecular profiling of the tumors, the failure to achieve endpoint objectives could possibly be due to the tumor heterogeneity amongst the patients. Varlitinib, a pan-HER inhibitor, also failed to meet primary endpoint objectives in CCA patients that failed first-line treatment [NCT02609958]. A clinical trial of bortezomib, proteasome inhibitor, was prematurely discontinued because of the lack of partial response [NCT00085410]. It is challenging to incorporate the tumor heterogeneity of the recruited patients in the study design. Additionally, without fully understanding the underlying mechanisms of the disease, there can be inconsistencies in tumor response when translated from in vitro to in vivo, and to the clinical setting. Therefore, inadequate stratification of patients can lead to inconclusive outcomes of the clinical trials.

Evidently, infigratinib (BGJ398), a selective FGFR inhibitor, has exhibited promising outcomes in a CCA patient cohort containing the *FGFR2* fusions. However, almost all patients eventually develop resistance due to acquired secondary mutations [35]. The evaluation of such clinical studies is based on tumor response and not directed towards understanding the underlying molecular mechanism of action of the drugs, this leads to the possibility of acquired resistance mechanism without any means to overcome the issue. Nevertheless, the urgency for accurate treatment for CCA patients is compelling and the need for targeted therapy, to treat different subsets of patients with distinct molecular signature, is imminent. Therefore, it is noteworthy that the FDA granted accelerated approval for pemigatinib, a novel FGFR inhibitor, to be used in treatment of CCA patients that are positive for *FGFR2* fusions and have failed first line chemotherapy [36] based on outcomes from a multi-cohort Phase II clinical trial [NCT04096417]. Currently, pemigatinib is in Phase III clinical trials as first-line treatment for CCA patients with *FGFR2* fusions. The accelerated approval by the FDA has expedited the drug development of pemigatinib and moved it towards clinical use. This is proof-of-concept that biomarker driven stratification of the patients results in a better outcome of clinical trials. Altogether, this suggests that the current clinical trial platform is lethargic in meeting the urgent need for novel therapeutics for CCA treatment, yet, when patients are accurately stratified and treated accordingly, there are improved outcomes in clinical trials.

Failed trials in HICs are not encouraged for further investigation in the LMICs, despite possible discrepancies in patient response to the drugs. Moreover, the clinical trials for CCA are designed and conducted based on the research of CCA patients in HICs. Due to this fact, many patients, particularly those in LMICs, are impeded from possibly effective therapies. Moreover, the rising costs of research and development discourage the LMICs to drive novel drugs for development, despite the growing demand. Hence, for effective management of CCA, research attention should be focused on driving more targeted therapy drugs towards approval. Consequently, this highlights that a more proficient system of clinical trials, which not only expedites the drug development process but also considers tumor heterogeneity and underlying mechanism of drug response, is needed to increase the chances of regulatory approval of targeted therapy drugs for CCA treatment.

## 4. What Are Co-Clinical Trials? 

The co-clinical project was first established as a platform for translational research in cancer to cure acute promyelocytic leukemia (APL) [37]. This platform utilizes the advancement of preclinical models, that can accurately replicate tumor heterogeneity, to stratify patients into treatable subtypes. The main objective of this platform is to fast track the development of drugs to practice precision oncology, so that treatment can be tailored to patients, individually [38]. The co-clinical trial platform is expected to reduce the disparity that exists between pre-clinical studies and clinical trials by conducting both the studies in parallel, in contrast to the sequential order in the conventional drug development process. 

**Figure 1:**
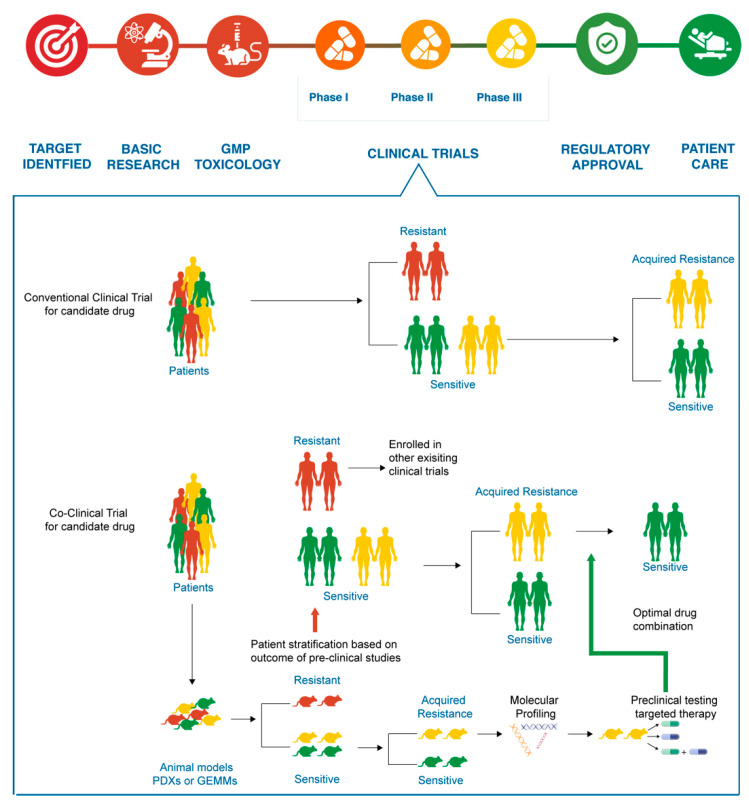
The conventional drug development process versus co-clinical trials. The different phases of clinical trials in the conventional drug development process take approximately 10–20 years for regulatory approval of the candidate drugs. Preclinical studies with animal models and clinical trials are conducted concurrently in co-clinical trials. The real-time data integration between the two parallel studies can accurately stratify patients into resistant or sensitive subtypes. The patients classified into resistant subtypes can be tested for enrolment in other existing clinical trials. The patient cohorts of the sensitive phenotype are then recruited to the trial with the animal study conducted in parallel. This will improve trial outcomes for candidate drugs and therefore encourage regulatory approval.

Currently, the drug development process for targeted therapies in CCA follows the conventional model which is both time-consuming and labor intensive. It roughly takes about 10–20 years for a candidate drug in its journey from the bench to the bedside [38] and only 13% of all drugs in clinical trials are FDA approved [39]. The schematics of co-clinical trials compared to conventional clinical trials is represented in Figure 1. 

The concept of co-clinical trial is to simultaneously conduct both human clinical trials and preclinical testing (also known as “mouse hospital”). Initially, patients that meet the criteria of the clinical study will be recruited, much like the initial stages of a conventional clinical trial. Subsequently, molecular profiling of the tumor tissues from the patients will identify the appropriate animal models to be used in the study, which will be conducted in parallel to the human clinical trials. For pre-clinical testing, the animal models are set up with a similar treatment, disease-monitoring and result acquisition protocols. The data from the pre-clinical studies are shared in real-time to inform drug response and resistance in patients in the clinical study. Patients can be stratified into subtypes based on the drug response from the representative animal models [37,38,40]. Hence, pre-clinical studies can inform outcomes of the clinical study so that it can be optimized, thereby improving outcomes of the clinical trials and enabling the discovery of potential therapies for cancer treatment.

## 5. Co-Clinical Trials to Accelerate Drug Development in CCA

Co-clinical trials are expected to improve drug development of targeted therapies in CCA by improving clinical trial outcomes. The initial drug screening in the pre-clinical studies, using animal models, allows for rapid stratifications of the patient population based on drug response and resistance. Patient population can be stratified into resistant or sensitive subtypes based on the outcomes from the pre-clinical studies. Patients with resistant subtypes can be removed from the study as they are unlikely to respond to that particular treatment, hence, this is expected to improve clinical trial outcomes for candidate drugs by limiting the patient cohort to the sensitive subtype. For the most part, candidate drugs fail to reproduce the tumor response in a clinical setting when translating from pre-clinical studies because they are performed separately. In co-clinical trials, the candidate drugs are tested in preclinical animal models, that represent the genetic subtypes of patients, using the same protocol that is to be used in the clinical trials. The results between both the studies are shared in real-time so that the treatment protocol can be adjusted and optimized to achieve the best possible outcomes [40]. This reduces the gap between preclinical research, clinical testing, and patient care by facilitating collaborative studies between academic and clinical researchers, thereby curbing the time taken for clinical trials [38]. The rapid stratification of patients into potential responders and non-responders based on experimental validation is expected to improve clinical trial outcomes and therefore expedite drug development in cancer. Therefore, co-clinical trials are expected to considerably improve the clinical trial outcomes in CCA and consequently, accelerate the drug development process and encourage more drugs to be available for treatment.

## 6. Proof-of-Concept of Co-Clinical Trials Expediting Drug Development in Other Cancers

Co-clinical trials were first established to cure APL, the results of this effort have been incredibly profound and have set the foundation for rethinking clinical studies. Researchers have utilized co-clinical trials (parallel testing using “mouse-hospitals) to optimize treatment for APL, the results of which have now been used to cure the disease [37]. The authors postulated that co-clinical trials could expedite the development of therapies in other cancers, provided that the infrastructure for real-time integration of data sharing between the pre-clinical and clinical studies is established. 

Co-clinical studies in several cancers have demonstrated that animal models can replicate sensitivity in clinical trials, predict resistance, and identify biomarkers that can predict sensitivity [41,42,43,44,45,46,47]. A summary of the studies is listed in Table 1. 

Co-clinical studies in lung and prostate cancers have already curbed the time taken for drug development from the bench-to-bedside to 3 years as opposed to the conventional 10–20 years [38]. A co-clinical study of dovitinib efficacy in patient-derived xenograft (PDX) models effectively replicated the drug response in Phase II clinical trials of lung squamous cell carcinoma patients. These results highlight FGFR pathway activation as biomarkers for predicting response to dovitinib [41]. Another study in lung cancer compared the efficacy of crizotinib (ALK inhibitor) against standard-of-care agents, Docetaxel or Pemetrexed, in an EML4-ALK mouse model and Phase III human clinical trials. The animal models efficiently predicted the response in human patients with EML4-ALK positive NSCLC and established the proof-of-concept of the co-clinical trial platform [42].

Therefore, it is warranted that the co-clinical trial platform stands to expedite drug development in CCA. The most challenging part in this system would be to establish a feasible timeframe for simultaneous collections and integration of results from both the patients and animal studies. Disease progression must be monitored at regular intervals with rapid and sensitive methods. This requires a multidisciplinary team of physicians, surgeons, radiologists, oncologists, and researchers to work cohesively and share knowledge in real-time. To implement co-clinical platform for effective management of CCA, accurate preclinical models that can replicate the tumor biology as well as an infrastructure to establish real-time data integration are required.

## 7. Animal Models Are Used as Pre-Clinical Models in the Current Co-Clinical Trial System

Accurate stratification of the patients is one of the key strategies in co-clinical trials to improve outcomes in the clinical trials. Animal models must accurately reproduce the patient response, so that their genetic profile can be used to stratify patients in the clinical study. This highlights the importance of using the right models in co-clinical trials to effectively improve trial outcomes for the candidate drugs.

Currently, only mouse models are used in co-clinical trials [38]. Animal models, typically patient-derived xenografts (PDX) or genetically engineered mouse models (GEMM), have been used in oncological research due to their close resemblance to the human biological system. PDXs continue to be the researcher’s favorite to investigate cancer, as they have shown insights into understanding the molecular mechanism of the disease [49,50,51,52]. In addition, tumor implants can be passaged and maintained for several generations [53]. The implanted tissues in PDXs are known for their ability to retain biological architecture and heterogeneity of the original tumors [51]. Furthermore, the size of the tumor in PDXs can be easily measured post-treatment for monitoring disease progression. PDX models have dependably replicated the potential outcome in Phase II co-clinical study of arsenic oxide in relapsed small cell lung cancer patients [44]. This makes PDXs the most trusted model of choice in cancer research and drug development process and consequently, in the co-clinical trial platform [40].

Moreover, researchers are using GEMMs to understand the biology of cancer driven by specific mutations [54]. The mouse models can be designed to include specific genetic mutations to investigate their role in disease progression and drug resistance. In CCA, this model can be particularly useful in understanding the implications of oncogenic drivers, such as *FGFR2* fusions. While treatment with FGFR inhibitors is effective in terms of tumor response, the patients ultimately develop drug resistance, possibly due to acquired secondary mutations [35]. Overall, the mouse models are scalable and ideal candidates for co-clinical trials. However, the limitation in using animal in vivo models is that it cannot accurately predict the drug efficacy in humans because the interaction within the stroma is different in the animals, and the use of immunosuppressed mice can mislead evaluation [40]. Additionally, animal studies have high accompanying costs and are labor intensive. Furthermore, the use of live subjects is a cause for ethical concern. The advantages and disadvantages of the different animal models are listed in detail in Table 2.

Nonetheless, PDXs and GEMMs are the most commonly used animal models in co-clinical trials. Research efforts into developing pre-clinical models for co-clinical trials using other animals, such as zebra fish and porcine models, are on-going [55,56]. Although, considering the ethical concerns regarding the use of animal models in research, the use of non-animal models may seem more lucrative to researchers. In addition, several studies have denoted the accuracy of non-animal models in predicting drug efficacy and toxicity. However, in CCA, considering the costs and ethical concerns that accompany animal models, we should contemplate the integration of non-animal models in the co-clinical trial platform.

## 8. Potential Use of Non-Animal Models as Pre-Clinical Models in Co-Clinical Trials for CCA

Advances in techniques in handling human tissues and cell-based models demonstrate the promise of using non-animal models in oncology. Further, 3D based cell culture assays with the use of organoids or spheroids can mimic the tumor microenvironment [57,58]. Micro-engineered systems incorporate the use of biomaterials to resemble the physiological setting to that of a human. It is more clinically relevant to humans than animal-based models due to the addition of human tumor microenvironment components. Microfluidics is applied to synthesize systems that resemble blood vessels. Lab-on-a-chip models can be used for drug development and screening [57,59,60,61]. It is scalable to test multiple drug conditions and portable and, eventually over time, cost-effective. Additionally, circulating tumor cells (CTCs) isolated from blood samples can be cultured and can be tested for drug sensitivity as in vitro predictive models [62]. 

CCA is highly heterogenous and patients are often diagnosed at advanced stages with varied tumor microenvironments, which means the preclinical testing models need to accurately replicate the physiological setting of each patient [63]. The advantages and disadvantages of potential non-animal models that may be expedient in co-clinical trials in CCA are listed in Table 3. 

The advantages of non-animal models are expected to overcome the limitations of animal models especially, the ethical concerns regarding inflicting pain on live subjects. However, researchers are reluctant to abandon animal models completely and switch to non-animal-based models due to reliability of results and extensive validation using animal models. While it is imperative to validate research outcomes in multiple systems, from in vitro to in vivo, this process of substantiation delays researchers from getting results. Furthermore, there is limited availability of data that can be extrapolated from databases to circumvent this issue. Yet, several studies have established proof-of-concept that patient-derived in vitro models can represent patient tumors in co-clinical trials in colorectal cancer [64], rhabdomyosarcoma [65], and rectal cancer [66]. While, the efforts for developing a dedicated CCA model capable for predictive pre-clinical testing is ongoing, there are numerous experimental models currently available for CCA research [63].

## 9. A Proposed Model of Co-Clinical Trials in CCA to Accelerate Drug Development

**Figure 2:**
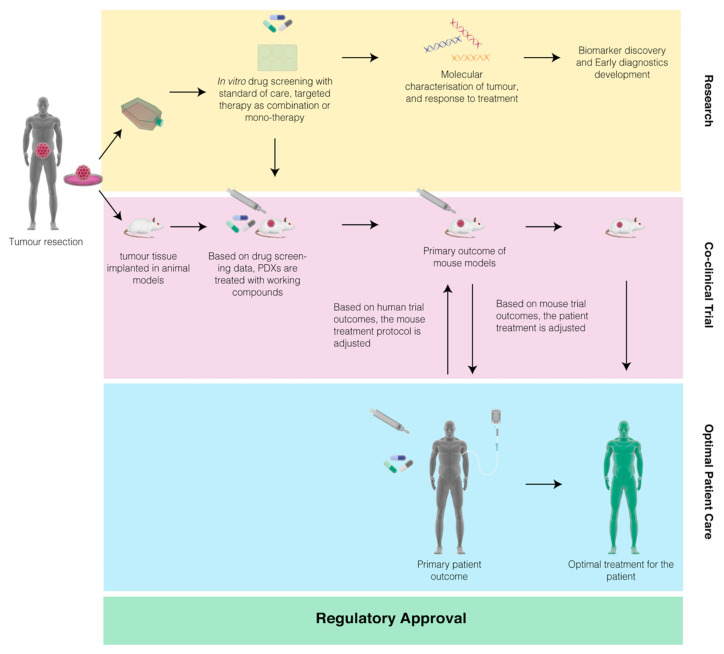
Schematics of co-clinical trials to expedite drug and research development in cholangiocarcinoma (CCA). In this proposed system, the patient is enrolled in the study after being diagnosed with CCA. Post-surgical resection, the CCA tissue is implanted in patient-derived-xenograft (PDX) models and derived into primary cell lines. If the tumor is unresectable, the molecular diagnosis of biopsies will enable researchers to design genetically engineered mouse models (GEMMs) and edited cell line models specific to the patients’ gene signature. The non-animal models are used for molecular profiling and initial drug screening with candidate drugs as monotherapy or in combination with other therapies. The animal models are then treated with prospective treatment protocol based on the initial drug screening. The treatment regime in the patient trials is then adjusted based on the real-time data integration from the animal studies, ensuring that each individual patient always receives optimal care.

All tumor models have with their limitations, therefore, with that consideration and also taking tumor heterogeneity into account, we propose a novel model of integrating co-clinical trials in CCA. We suggest that a preliminary drug screening using a pre-clinical in vitro model, such as organoids or spheroids, should be conducted prior to the parallel testing in pre-clinical animal models and human patients. The schematic representation of this proposed system is depicted in Figure 2. 

In this system, firstly, molecular profiling of the patient is performed to select the appropriate animal model for the co-clinical study. Secondly, before the initiation of either the animal or human study, a preliminary screening of candidate targeted therapy drugs as either mono therapy or in combinations with chemotherapy will be conducted in non-animal models. Thirdly, only the most effective treatment conditions will be used to treat the patient and animal models, in parallel, for the co-clinical study. PDX models will be used for patients that undergo surgical resection and GEMMs will be used for patients that have unresectable tumor. Finally, based on the response of animal studies to the candidate drugs, the patients in the human study can be stratified into responders and non-responders. This means that the treatment regime is tested in two preclinical systems using both, animal and non-animal models. Therefore, it is optimized for each individual and this ensures that drugs in clinical trials, particularly targeted therapies, will have better chances of meeting their endpoint objectives. Hence, this will facilitate the availability of many targeted therapies for CCA treatment. In addition, the inclusion of non-animal models will reduce costs related to animal handling by reducing the numbers of models used, which will be beneficial for researchers. 

While this proposed framework has been designed to facilitate recruitment of CCA patients with resectable tumor, in the true nature of clinical trials, the research design can be modified to cater to other patients with unresectable tumors. For example, in clinical trials with specific molecular targets, GEMM models can be developed to include those targets and genetically edited organoid cultures can be developed using CRISPR/Cas9 techniques to represent such CCA patients [63]. In Thailand, there are many centers that conduct multiple clinical trials for CCA, investigators recruit patients that fail first line standard-of-care to the different studies as they deem appropriate. With the implementation of co-clinical trials, physicians can test trial drugs from the current studies in the non-animal model and based on the response and molecular characterization of the tumors, they can rapidly stratify patients and then enroll them in the appropriate co-clinical study. As tumor heterogeneity is a prime characteristic of CCA, the integration of the co-clinical platform will facilitate drug development in CCA by improving clinical trial outcomes. Hence, it is self-evident that people with CCA will benefit from this initiative.

## 10. Applications of Co-Clinical Trial in CCA Research and Development 

The benefits of co-clinical trials are not only limited to accelerating the drug development process, but it is also advantageous in research and development in CCA by addressing many of the existing knowledge gaps. Furthermore, the implementation of co-clinical trials for CCA will establish a platform for translational research, as this will increase the availability and access to tissues and blood samples from patients enrolled in the co-clinical study to establish tumor tissue biobanks. Researchers can learn more about the CCA pathogenesis and therefore develop relevant models for precision medicine and characterize CCA tissues based on molecular signatures. 

Through a cohort study, the heterogeneity and molecular profile of CCA may become clearer, which can help identify key biomarkers that can predict drug response and resistance. Researchers can gain insight into the underlying molecular mechanisms required by particular drugs to elicit a response or develop acquired resistance. This allows drug developers to improve the future generations of therapeutic compounds accordingly [38]. The co-clinical trial platform offers a multidisciplinary approach in understanding and managing CCA.

## 11. Discussion

Co-clinical trials will expedite drug development by improving clinical trial outcomes in CCA by reducing the disparity between trials in pre-clinical models and in patients. Perhaps the establishment of this platform will be the most challenging, however, once established, the infrastructure will enable researchers, oncologists, radiologists, and surgeons to work in parallel to inform appropriate treatment in patients to optimize trial outcomes. In addition to drug development, real-time integration of data from the clinical and co-clinical study can help design and develop early detection and companion diagnostics for CCA. Researchers will be able to adopt a multidisciplinary approach for CCA management. 

It is widely acknowledged that the biggest challenge in CCA management is understanding the biology of the cancer [4,67]. One of the prime limitations of clinical trials for CCA is the constraint in study design. Patients are often recruited as a mixed cohort, without the consideration of tumor heterogeneity, therefore, clinical studies involving targeted therapies tend to fail. Whereas, in co-clinical trials, not only are the molecular targets taken into account, there is also flexibility in the study design. Because each trial patient is treated as an individual, each patient receives optimal care. The implementation of co-clinical trials is anticipated to improve clinical trial outcomes, thereby increasing the number of targeted therapy drugs available for treatment and allowing physicians to practice precision medicine. There are currently a number of different targeted therapies investigated against *ERBB2* [NCT03602079, NCT04466891], *FGFR* [NCT03773302, NCT02150967, NCT03656536, NCT02150967, NCT03230318], *IDH* [NCT03212274, NCT03878095, NCT03991832, NCT04521686], and *ROS1/ALK* [NCT02568267] as either monotherapies or in combination with chemotherapy in clinical trials in CCA. These clinical trials can benefit from the integration of the co-clinical platform. 

The implementation of the co-clinical trial platform is feasible now more than ever as the concept of precision medicine in oncology is gaining momentum, especially in Thailand, with the molecular profiling of tumors as well as high-throughput drug screening using 3D cell culture models [68]. There are also a number of different in vitro and in vivo experimental models available for testing, exclusively, in CCA [63]. Furthermore, the Cholangiocarcinoma Screening and Care Program (CASCAP), an initiative by Khon Khaen University and Cholangiocarcinoma Foundation, is a healthcare program for CCA patients. The clinical data collected from this investigation have been stored in an extensive database complete with long-term follow-up of the patients undergoing different medical interventions [69]. Moreover, the Newton Fund initiatives have enabled experts from multiple disciplines to collaborate and drive towards implementing an infrastructure for co-clinical trials. Together, this ensures a comprehensive foundation required to initiate co-clinical trials in Thailand.

Therefore, this infrastructure can be utilized for implementing co-clinical trials, which will facilitate drug development in CCA. While the initial cost and time for setting up the infrastructure and expertise may be the prime drawback of implementing this system, it should not discourage us from taking a step towards progress. The benefits of co-clinical trials outweigh the costs in the long run. A large number of patients with progressive diseases from low-income countries can benefit from this initiative. Nonetheless, there are many further studies required to streamline this process, still, it stands to reason that co-clinical trials may be the future of precision medicine in cancers including cholangiocarcinoma. 

## Figures and Tables

**Table 1:** Co-clinical trials in cancer.

Cancer Type	Testing Model	Drug	Outcome	Reference
Lung squamous cell carcinoma	PDX	dovitinib	FGFR pathways can predict the sensitivity to dovitinib both in cell lines and PDX tumors.	[41]
Non-small cell lung cancer	GEMM	crizotinib	ALK inhibitor crizotinib was more effective than standard-of-care drugs in a comparative co-clinical study with patients and GEMM mouse models. The study facilitates the prediction of crizotinib resistance.	[42]
*KRAS* mutant non-small cell lung cancer	GEMM	selumetinib	The addition of selumetinib proved differential response in mice with lung cancer caused by Kras mutation. Mice with *Kras* + *p53* mutations were sensitive but mice with *Kras* + *Lkb1* mutations showed resistance to combination therapy.	[43]
Relapsed small cell lung cancer	PDX	arsenic trioxide	Strong correlation between the response of arsenic trioxide and cisplatin in SCLC clinical and PDX model supports the use of PDX models to screen promising anticancer agents prior to clinical testing in patients.	[44]
Prostate cancer	GEMM	androgen deprivation therapy	Genetic mouse models along with patients elucidate the mechanism of castration resistance. Results encourage the stratification of patients based for precision medicine.	[45]
Prostate cancer	GEMM	5α-reductase inhibitors	Genetic expression profiling of NKX3.1 mutant mouse models and patients in treatment were compared NKX3.1 expression predicts response to 5α-reductase inhibitors (5-ARI).	[46]
Melanoma	GEMM	BRAF inhibitor	Co-clinical analysis of human and mouse melanomas elucidates the patterns of resistance to BRAF inhibitors. This study also identifies biomarkers to predict response.	[47]
Head and neck cancer	PDX	buparlisib	Integrated mouse trials helped identify the anti-tumor effects of combination therapy with cetuximab and revise the treatment regime, thus enabling promising outcomes of the clinical trials.	[48]

**Table 2:** Advantages and disadvantages of animal models in co-clinical trial.

Model	Advantages	Disadvantages	References
PDX	Most reliable model in cancer studies. Currently the gold-standard for preclinical drug efficacy and toxicity studies in the drug regulatory process. PDX implants retain heterogeneity and architecture of the tumor. PDX implants can be passaged and therefore maintained to test with multiple therapy formats. Therapy induced cancer progression can be observed by measuring tumor size. Scalable for co-clinical studies.	Variable success rate of engrafting depending on the tumors which results in selective bias. Relevance of tissue-specific results as the tissues are only engrafted subcutaneously in mice and in the orthotropic site. Variable sensitivity to treatments by human and mouse CAFs. Ethical concerns regarding inflicting pain on live subjects. Immuno-compromised mice are used, and immune-cell evaluation cannot be determined. Labor intensive and technically difficult and financially challenging.	[40,51,52,54]
GEMM	Distinctive genetic modifications that drive cancer progression and development can be focused. Scalable for co-clinical studies.	Labor intensive and technical difficult and financially challenging. Development of genetically engineered mouse models are expensive. They have also been known to show different responses to immune mediators when compared with human. Bias, as it cannot replicate the heterogeneity in the system. Requirement of enrolling multiple genetic models in one holistic study.	[54]

**Table 3:** Advantages and disadvantages of non-animal models in co-clinical trials.

Model	Advantages	Disadvantages	References
3D Cell culture (e.g., organoids and spheroids)	Scalable for co-clinical studies, multiple single agents, and in combinations can be studied. Organoids and spheroids can mimic the 3D architecture of tumor microenvironment. Real-time biomarker imaging for drug response through high-throughput single cell imaging. Cost-effective in the long run. No ethical concerns regarding inflicting pain on animals. Increasing biobank tissue collection with accompanying clinical metadata will help with this process.	Logistics transport and storage. Technically challenging to process and maintain viable tissues. Setting up a new system utilizes more time and money. Reluctance of researchers to abandon old systems. Lack of previous data comparability.	[58,59,64]
Micro-engineered systems	Lab-on-a chip models that mimic multiple organ system can also imitate the systemic nature of humans in vitro. Mimics metastatic environment. Retain genotype and phenotype. No ethical concerns, regarding animal handling. Easy to use. Scalable for drug screening. Reduced costs, hence, fewer consumables and sample volume. Improved prediction of pre-clinical assays. Integration of culture system in cell-based assays (CTCs)	Scalable but initial costs will be high. High capital investment. Fewer experiment data available to correlate and validate this system.	[61]

## Data Availability

Not applicable.
